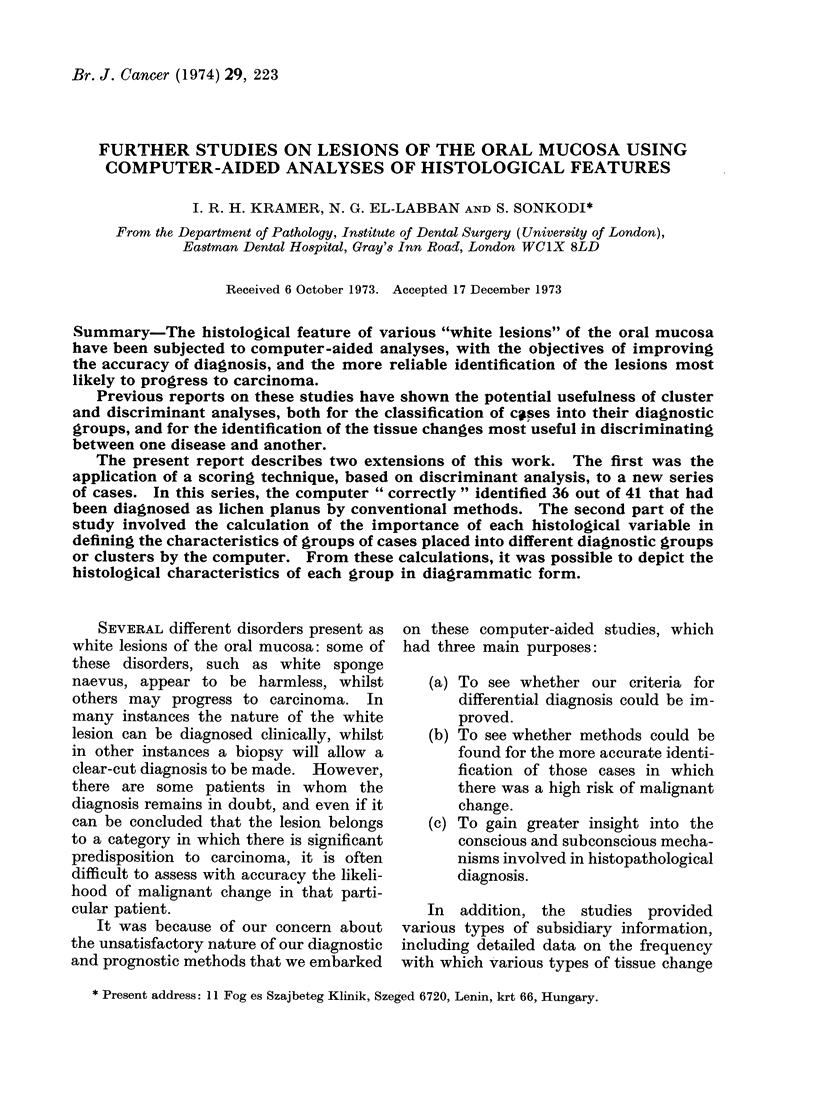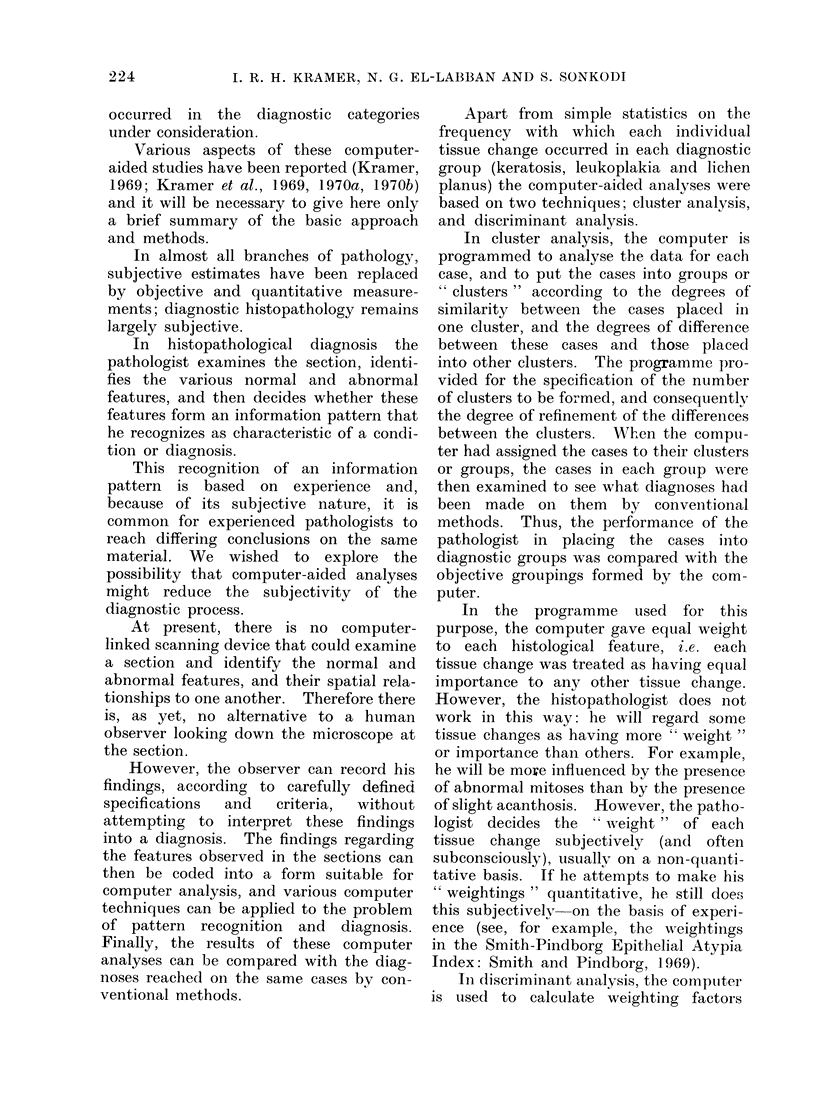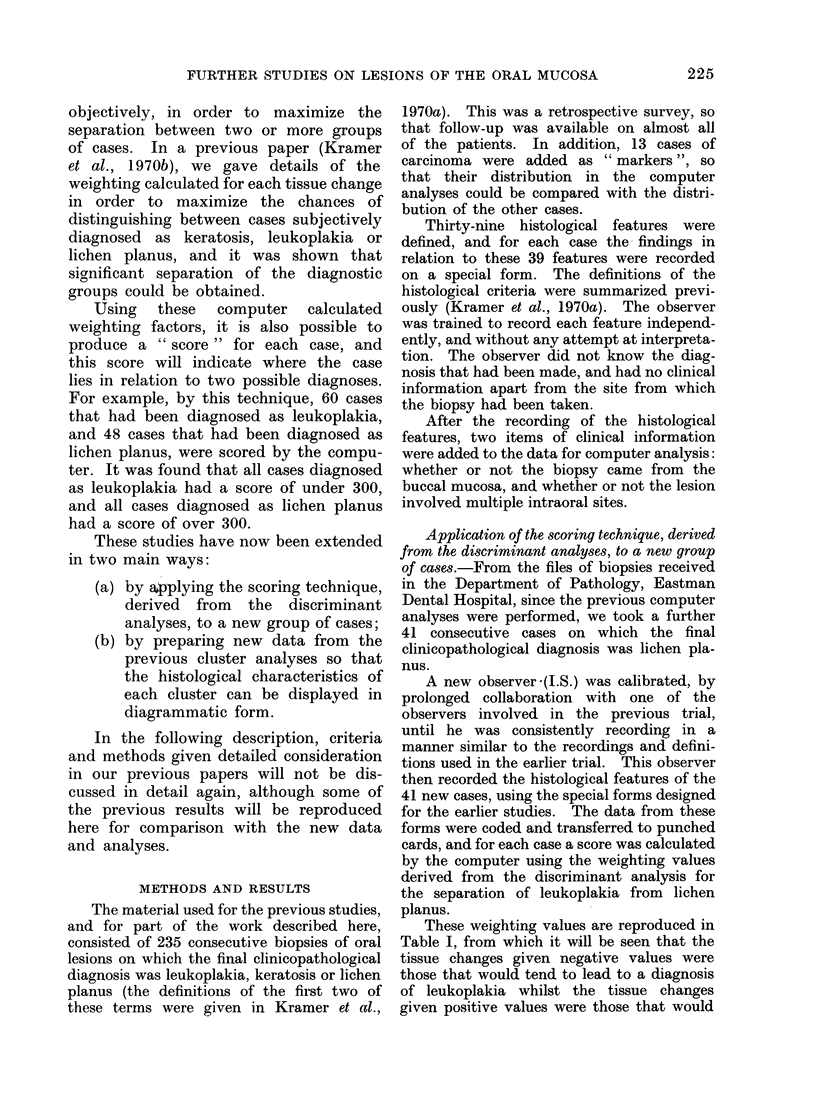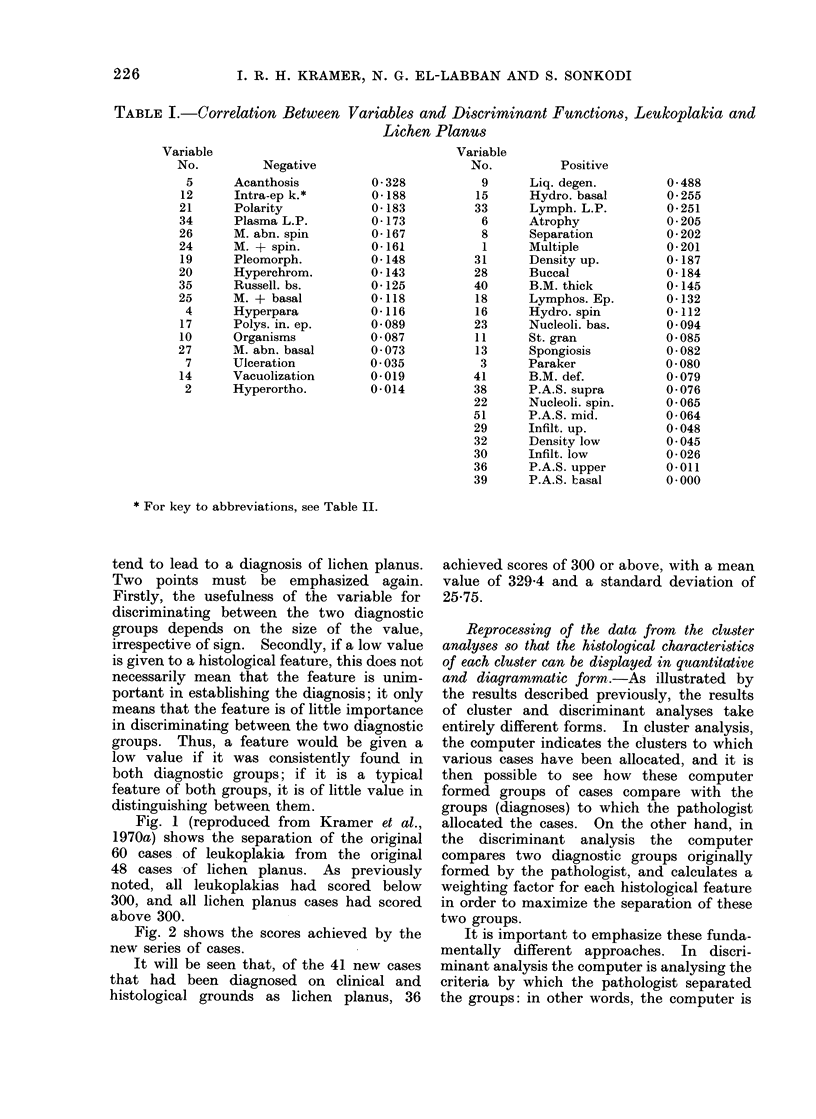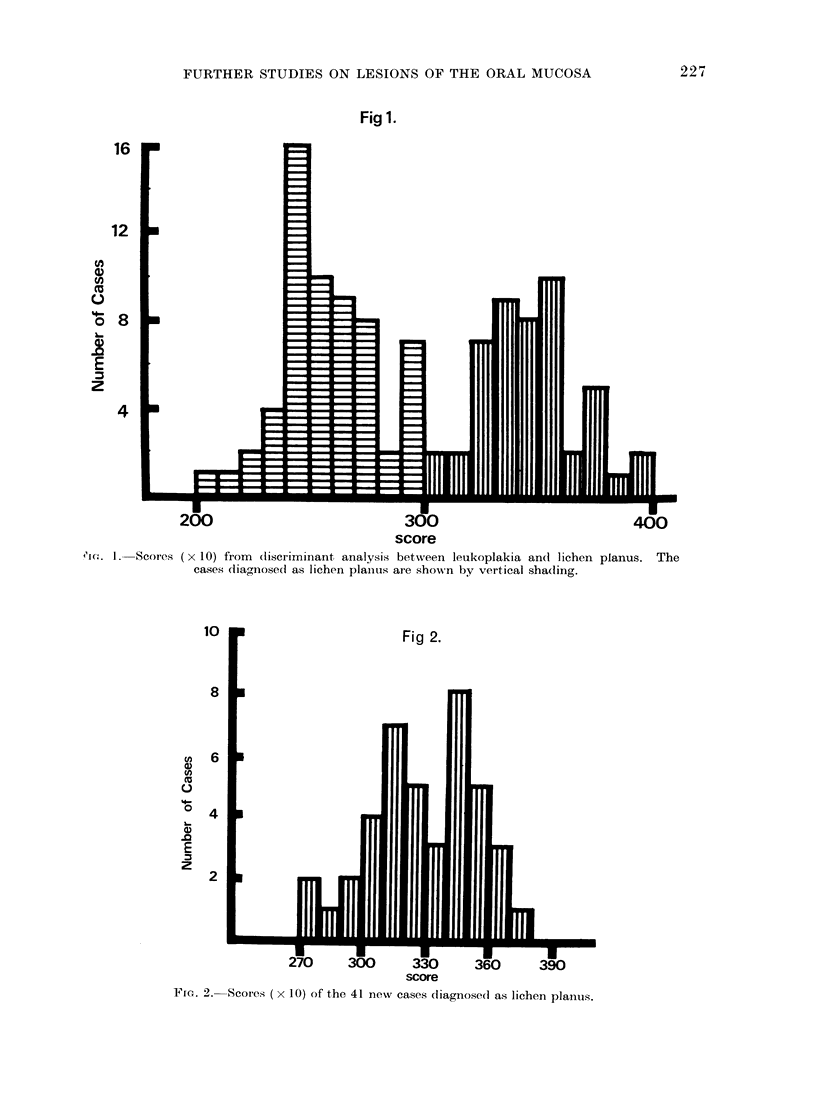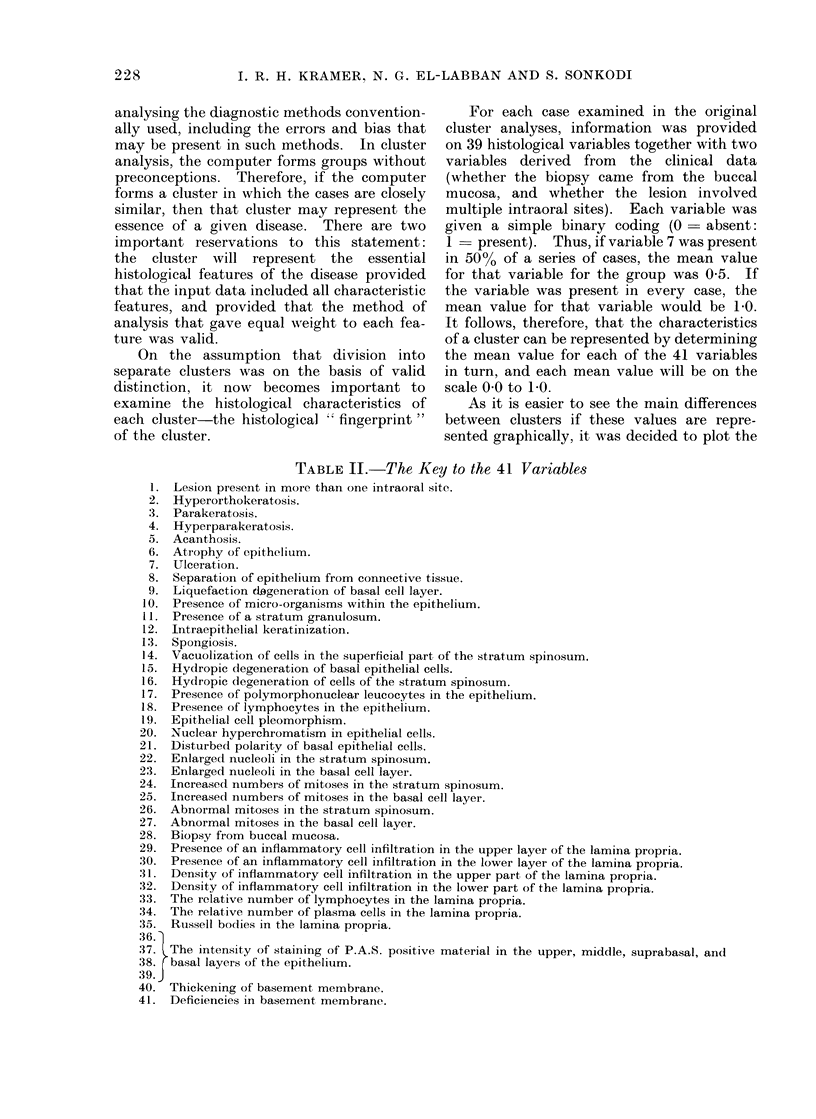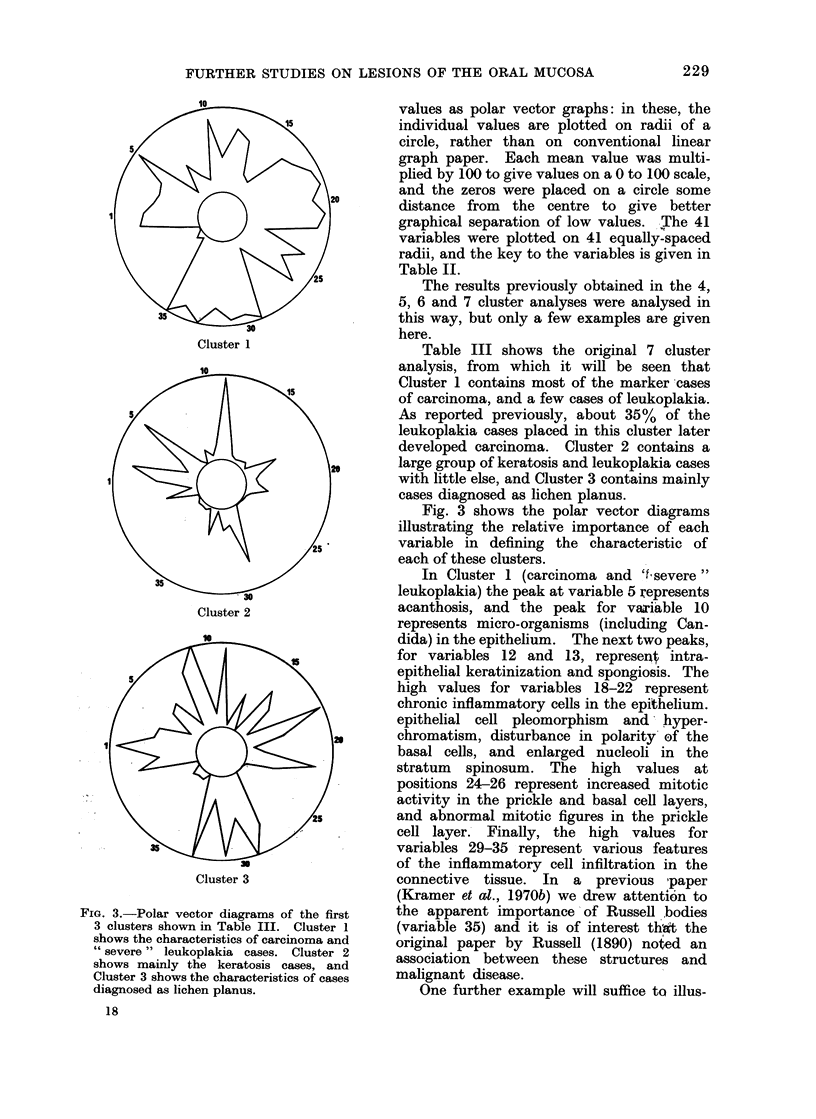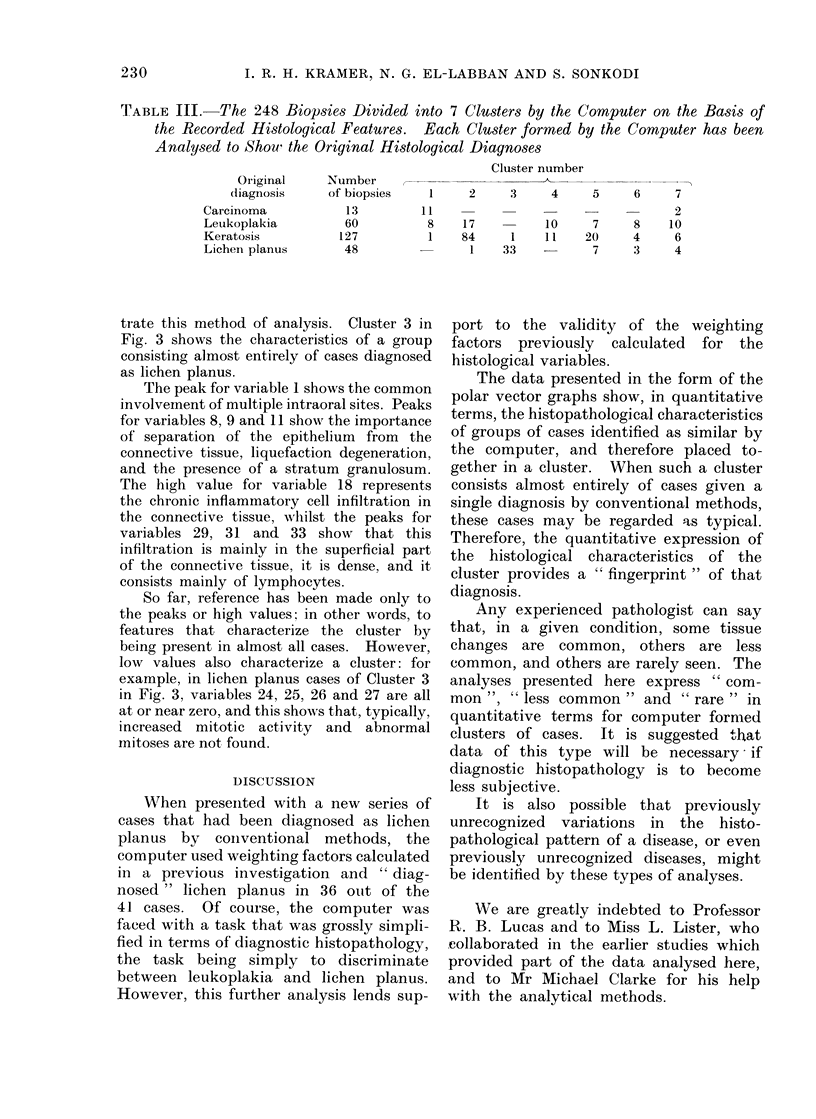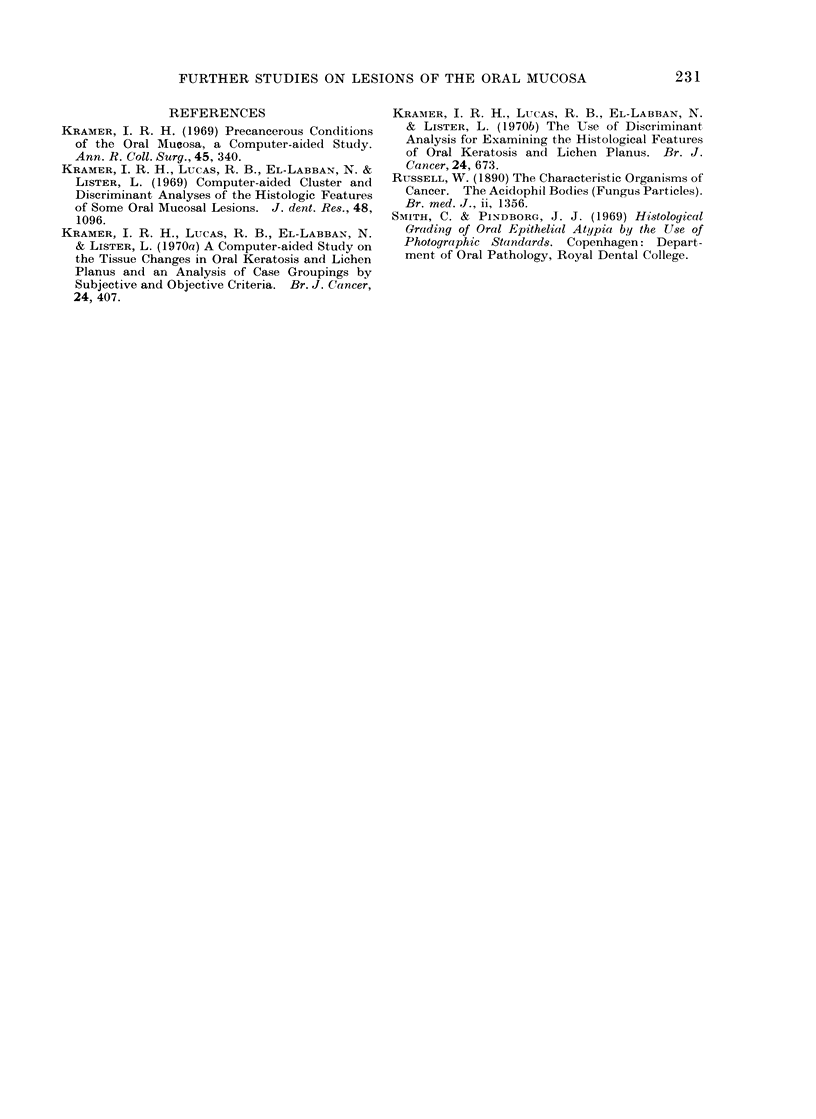# Further Studies on Lesions of the Oral Mucosa Using Computer Aided Analyses of Histological Features

**DOI:** 10.1038/bjc.1974.61

**Published:** 1974-03

**Authors:** I. R. H. Kramer, N. G. El-Labban, S. Sonkodi

## Abstract

The histological feature of various “white lesions” of the oral mucosa have been subjected to computer-aided analyses, with the objectives of improving the accuracy of diagnosis, and the more reliable identification of the lesions most likely to progress to carcinoma.

Previous reports on these studies have shown the potential usefulness of cluster and discriminant analyses, both for the classification of cases into their diagnostic groups, and for the identification of the tissue changes most useful in discriminating between one disease and another.

The present report describes two extensions of this work. The first was the application of a scoring technique, based on discriminant analysis, to a new series of cases. In this series, the computer “correctly” identified 36 out of 41 that had been diagnosed as lichen planus by conventional methods. The second part of the study involved the calculation of the importance of each histological variable in defining the characteristics of groups of cases placed into different diagnostic groups or clusters by the computer. From these calculations, it was possible to depict the histological characteristics of each group in diagrammatic form.


*Br. J. Cancer* (1974) **29**, 223

### FURTHER STUDIES ON LESIONS OF THE ORAL MUCOSA USING COMPUTER-AIDED ANALYSES OF HISTOLOGICAL FEATURES

I. R. H. KRAMER, N. G. EL-LABBAN AND S. SONKODI*

*From the Department of Pathology, Institute of Dental Surgery (University of London), Eastman Dental Hospital, Gray's Inn Road, London WC1X 8LD*

Received 6 October 1973. Accepted 17 December 1973

**Summary—The histological feature of various "white lesions" of the oral mucosa have been subjected to computer-aided analyses, with the objectives of improving the accuracy of diagnosis, and the more reliable identification of the lesions most likely to progress to carcinoma.**

**Previous reports on these studies have shown the potential usefulness of cluster and discriminant analyses, both for the classification of cases into their diagnostic groups, and for the identification of the tissue changes most useful in discriminating between one disease and another.**

**The present report describes two extensions of this work. The first was the application of a scoring technique, based on discriminant analysis, to a new series of cases. In this series, the computer " correctly " identified 36 out of 41 that had been diagnosed as lichen planus by conventional methods. The second part of the study involved the calculation of the importance of each histological variable in defining the characteristics of groups of cases placed into different diagnostic groups or clusters by the computer. From these calculations, it was possible to depict the histological characteristics of each group in diagrammatic form.**

SEVERAL different disorders present as white lesions of the oral mucosa: some of these disorders, such as white sponge naevus, appear to be harmless, whilst others may progress to carcinoma. In many instances the nature of the white lesion can be diagnosed clinically, whilst in other instances a biopsy will allow a clear-cut diagnosis to be made. However, there are some patients in whom the diagnosis remains in doubt, and even if it can be concluded that the lesion belongs to a category in which there is significant predisposition to carcinoma, it is often difficult to assess with accuracy the likelihood of malignant change in that particular patient.

It was because of our concern about the unsatisfactory nature of our diagnostic and prognostic methods that we embarked on these computer-aided studies, which had three main purposes:

(a) To see whether our criteria for differential diagnosis could be improved.
(b) To see whether methods could be found for the more accurate identification of those cases in which there was a high risk of malignant change.
(c) To gain greater insight into the conscious and subconscious mechanisms involved in histopathological diagnosis.

In addition, the studies provided various types of subsidiary information, including detailed data on the frequency with which various types of tissue change

* Present address: 11 Fog es Szajbeteg Klinik, Szeged 6720, Lenin, krt 66, Hungary.

occurred in the diagnostic categories under consideration.

Various aspects of these computer-aided studies have been reported (Kramer, 1969; Kramer *et al.*, 1969, 1970*a*, 1970*b*) and it will be necessary to give here only a brief summary of the basic approach and methods.

In almost all branches of pathology, subjective estimates have been replaced by objective and quantitative measurements; diagnostic histopathology remains largely subjective.

In histopathological diagnosis the pathologist examines the section, identifies the various normal and abnormal features, and then decides whether these features form an information pattern that he recognizes as characteristic of a condition or diagnosis.

This recognition of an information pattern is based on experience and, because of its subjective nature, it is common for experienced pathologists to reach differing conclusions on the same material. We wished to explore the possibility that computer-aided analyses might reduce the subjectivity of the diagnostic process.

At present, there is no computer-linked scanning device that could examine a section and identify the normal and abnormal features, and their spatial relationships to one another. Therefore there is, as yet, no alternative to a human observer looking down the microscope at the section.

However, the observer can record his findings, according to carefully defined specifications and criteria, without attempting to interpret these findings into a diagnosis. The findings regarding the features observed in the sections can then be coded into a form suitable for computer analysis, and various computer techniques can be applied to the problem of pattern recognition and diagnosis. Finally, the results of these computer analyses can be compared with the diagnoses reached on the same cases by conventional methods.

Apart from simple statistics on the frequency with which each individual tissue change occurred in each diagnostic group (keratosis, leukoplakia and lichen planus) the computer-aided analyses were based on two techniques; cluster analysis, and discriminant analysis.

In cluster analysis, the computer is programmed to analyse the data for each case, and to put the cases into groups or " clusters " according to the degrees of similarity between the cases placed in one cluster, and the degrees of difference between these cases and those placed into other clusters. The programme provided for the specification of the number of clusters to be formed, and consequently the degree of refinement of the differences between the clusters. When the computer had assigned the cases to their clusters or groups, the cases in each group were then examined to see what diagnoses had been made on them by conventional methods. Thus, the performance of the pathologist in placing the cases into diagnostic groups was compared with the objective groupings formed by the computer.

In the programme used for this purpose, the computer gave equal weight to each histological feature, *i.e.* each tissue change was treated as having equal importance to any other tissue change. However, the histopathologist does not work in this way: he will regard some tissue changes as having more " weight " or importance than others. For example, he will be more influenced by the presence of abnormal mitoses than by the presence of slight acanthosis. However, the pathologist decides the " weight " of each tissue change subjectively (and often subconsciously), usually on a non-quantitative basis. If he attempts to make his " weightings " quantitative, he still does this subjectively—on the basis of experience (see, for example, the weightings in the Smith-Pindborg Epithelial Atypia Index: Smith and Pindborg, 1969).

In discriminant analysis, the computer is used to calculate weighting factors objectively, in order to maximize the separation between two or more groups of cases. In a previous paper (Kramer *et al.*, 1970*b*), we gave details of the weighting calculated for each tissue change in order to maximize the chances of distinguishing between cases subjectively diagnosed as keratosis, leukoplakia or lichen planus, and it was shown that significant separation of the diagnostic groups could be obtained.

Using these computer calculated weighting factors, it is also possible to produce a " score " for each case, and this score will indicate where the case lies in relation to two possible diagnoses. For example, by this technique, 60 cases that had been diagnosed as leukoplakia, and 48 cases that had been diagnosed as lichen planus, were scored by the computer. It was found that all cases diagnosed as leukoplakia had a score of under 300, and all cases diagnosed as lichen planus had a score of over 300.

These studies have now been extended in two main ways:

(a) by applying the scoring technique, derived from the discriminant analyses, to a new group of cases;
(b) by preparing new data from the previous cluster analyses so that the histological characteristics of each cluster can be displayed in diagrammatic form.

In the following description, criteria and methods given detailed consideration in our previous papers will not be discussed in detail again, although some of the previous results will be reproduced here for comparison with the new data and analyses.

### METHODS AND RESULTS

The material used for the previous studies, and for part of the work described here, consisted of 235 consecutive biopsies of oral lesions on which the final clinicopathological diagnosis was leukoplakia, keratosis or lichen planus (the definitions of the first two of these terms were given in Kramer *et al.*, 1970*a*). This was a retrospective survey, so that follow-up was available on almost all of the patients. In addition, 13 cases of carcinoma were added as " markers ", so that their distribution in the computer analyses could be compared with the distribution of the other cases.

Thirty-nine histological features were defined, and for each case the findings in relation to these 39 features were recorded on a special form. The definitions of the histological criteria were summarized previously (Kramer *et al.*, 1970*a*). The observer was trained to record each feature independently, and without any attempt at interpretation. The observer did not know the diagnosis that had been made, and had no clinical information apart from the site from which the biopsy had been taken.

After the recording of the histological features, two items of clinical information were added to the data for computer analysis: whether or not the biopsy came from the buccal mucosa, and whether or not the lesion involved multiple intraoral sites.

*Application of the scoring technique, derived from the discriminant analyses, to a new group of cases.*—From the files of biopsies received in the Department of Pathology, Eastman Dental Hospital, since the previous computer analyses were performed, we took a further 41 consecutive cases on which the final clinicopathological diagnosis was lichen planus.

A new observer (I.S.) was calibrated, by prolonged collaboration with one of the observers involved in the previous trial, until he was consistently recording in a manner similar to the recordings and definitions used in the earlier trial. This observer then recorded the histological features of the 41 new cases, using the special forms designed for the earlier studies. The data from these forms were coded and transferred to punched cards, and for each case a score was calculated by the computer using the weighting values derived from the discriminant analysis for the separation of leukoplakia from lichen planus.

These weighting values are reproduced in Table I, from which it will be seen that the tissue changes given negative values were those that would tend to lead to a diagnosis of leukoplakia whilst the tissue changes given positive values were those that would

TABLE I.—*Correlation Between Variables and Discriminant Functions, Leukoplakia and Lichen Planus*

| Variable No. | Negative | | Variable No. | Positive | |
|---|---|---|---|---|---|
| 5 | Acanthosis | 0·328 | 9 | Liq. degen. | 0·488 |
| 12 | Intra-ep k.* | 0·188 | 15 | Hydro. basal | 0·255 |
| 21 | Polarity | 0·183 | 33 | Lymph. L.P. | 0·251 |
| 34 | Plasma L.P. | 0·173 | 6 | Atrophy | 0·205 |
| 26 | M. abn. spin | 0·167 | 8 | Separation | 0·202 |
| 24 | M. + spin. | 0·161 | 1 | Multiple | 0·201 |
| 19 | Pleomorph. | 0·148 | 31 | Density up. | 0·187 |
| 20 | Hyperchrom. | 0·143 | 28 | Buccal | 0·184 |
| 35 | Russell. bs. | 0·125 | 40 | B.M. thick | 0·145 |
| 25 | M. + basal | 0·118 | 18 | Lymphos. Ep. | 0·132 |
| 4 | Hyperpara | 0·116 | 16 | Hydro. spin | 0·112 |
| 17 | Polys. in. ep. | 0·089 | 23 | Nucleoli. bas. | 0·094 |
| 10 | Organisms | 0·087 | 11 | St. gran | 0·085 |
| 27 | M. abn. basal | 0·073 | 13 | Spongiosis | 0·082 |
| 7 | Ulceration | 0·035 | 3 | Paraker | 0·080 |
| 14 | Vacuolization | 0·019 | 41 | B.M. def. | 0·079 |
| 2 | Hyperortho. | 0·014 | 38 | P.A.S. supra | 0·076 |
| | | | 22 | Nucleoli. spin. | 0·065 |
| | | | 51 | P.A.S. mid. | 0·064 |
| | | | 29 | Infilt. up. | 0·048 |
| | | | 32 | Density low | 0·045 |
| | | | 30 | Infilt. low | 0·026 |
| | | | 36 | P.A.S. upper | 0·011 |
| | | | 39 | P.A.S. basal | 0·000 |

* For key to abbreviations, see Table II.

tend to lead to a diagnosis of lichen planus. Two points must be emphasized again. Firstly, the usefulness of the variable for discriminating between the two diagnostic groups depends on the size of the value, irrespective of sign. Secondly, if a low value is given to a histological feature, this does not necessarily mean that the feature is unimportant in establishing the diagnosis; it only means that the feature is of little importance in discriminating between the two diagnostic groups. Thus, a feature would be given a low value if it was consistently found in both diagnostic groups; if it is a typical feature of both groups, it is of little value in distinguishing between them.

Fig. 1 (reproduced from Kramer *et al.*, 1970*a*) shows the separation of the original 60 cases of leukoplakia from the original 48 cases of lichen planus. As previously noted, all leukoplakias had scored below 300, and all lichen planus cases had scored above 300.

Fig. 2 shows the scores achieved by the new series of cases.

It will be seen that, of the 41 new cases that had been diagnosed on clinical and histological grounds as lichen planus, 36 achieved scores of 300 or above, with a mean value of 329·4 and a standard deviation of 25·75.

*Reprocessing of the data from the cluster analyses so that the histological characteristics of each cluster can be displayed in quantitative and diagrammatic form.*—As illustrated by the results described previously, the results of cluster and discriminant analyses take entirely different forms. In cluster analysis, the computer indicates the clusters to which various cases have been allocated, and it is then possible to see how these computer formed groups of cases compare with the groups (diagnoses) to which the pathologist allocated the cases. On the other hand, in the discriminant analysis the computer compares two diagnostic groups originally formed by the pathologist, and calculates a weighting factor for each histological feature in order to maximize the separation of these two groups.

It is important to emphasize these fundamentally different approaches. In discriminant analysis the computer is analysing the criteria by which the pathologist separated the groups: in other words, the computer is

FIG. 1.—Scores (× 10) from discriminant analysis between leukoplakia and lichen planus. The cases diagnosed as lichen planus are shown by vertical shading.

FIG. 2.—Scores (× 10) of the 41 new cases diagnosed as lichen planus.

analysing the diagnostic methods conventionally used, including the errors and bias that may be present in such methods. In cluster analysis, the computer forms groups without preconceptions. Therefore, if the computer forms a cluster in which the cases are closely similar, then that cluster may represent the essence of a given disease. There are two important reservations to this statement: the cluster will represent the essential histological features of the disease provided that the input data included all characteristic features, and provided that the method of analysis that gave equal weight to each feature was valid.

On the assumption that division into separate clusters was on the basis of valid distinction, it now becomes important to examine the histological characteristics of each cluster—the histological "fingerprint" of the cluster.

For each case examined in the original cluster analyses, information was provided on 39 histological variables together with two variables derived from the clinical data (whether the biopsy came from the buccal mucosa, and whether the lesion involved multiple intraoral sites). Each variable was given a simple binary coding (0 = absent: 1 = present). Thus, if variable 7 was present in 50% of a series of cases, the mean value for that variable for the group was 0·5. If the variable was present in every case, the mean value for that variable would be 1·0. It follows, therefore, that the characteristics of a cluster can be represented by determining the mean value for each of the 41 variables in turn, and each mean value will be on the scale 0·0 to 1·0.

As it is easier to see the main differences between clusters if these values are represented graphically, it was decided to plot the

TABLE II.—*The Key to the* 41 *Variables*

1. Lesion present in more than one intraoral site.
2. Hyperorthokeratosis.
3. Parakeratosis.
4. Hyperparakeratosis.
5. Acanthosis.
6. Atrophy of epithelium.
7. Ulceration.
8. Separation of epithelium from connective tissue.
9. Liquefaction degeneration of basal cell layer.
10. Presence of micro-organisms within the epithelium.
11. Presence of a stratum granulosum.
12. Intraepithelial keratinization.
13. Spongiosis.
14. Vacuolization of cells in the superficial part of the stratum spinosum.
15. Hydropic degeneration of basal epithelial cells.
16. Hydropic degeneration of cells of the stratum spinosum.
17. Presence of polymorphonuclear leucocytes in the epithelium.
18. Presence of lymphocytes in the epithelium.
19. Epithelial cell pleomorphism.
20. Nuclear hyperchromatism in epithelial cells.
21. Disturbed polarity of basal epithelial cells.
22. Enlarged nucleoli in the stratum spinosum.
23. Enlarged nucleoli in the basal cell layer.
24. Increased numbers of mitoses in the stratum spinosum.
25. Increased numbers of mitoses in the basal cell layer.
26. Abnormal mitoses in the stratum spinosum.
27. Abnormal mitoses in the basal cell layer.
28. Biopsy from buccal mucosa.
29. Presence of an inflammatory cell infiltration in the upper layer of the lamina propria.
30. Presence of an inflammatory cell infiltration in the lower layer of the lamina propria.
31. Density of inflammatory cell infiltration in the upper part of the lamina propria.
32. Density of inflammatory cell infiltration in the lower part of the lamina propria.
33. The relative number of lymphocytes in the lamina propria.
34. The relative number of plasma cells in the lamina propria.
35. Russell bodies in the lamina propria.

36.–39. The intensity of staining of P.A.S. positive material in the upper, middle, suprabasal, and basal layers of the epithelium.

40. Thickening of basement membrane.
41. Deficiencies in basement membrane.

Cluster 1

Cluster 2

Cluster 3

FIG. 3.—Polar vector diagrams of the first 3 clusters shown in Table III. Cluster 1 shows the characteristics of carcinoma and " severe " leukoplakia cases. Cluster 2 shows mainly the keratosis cases, and Cluster 3 shows the characteristics of cases diagnosed as lichen planus.

values as polar vector graphs: in these, the individual values are plotted on radii of a circle, rather than on conventional linear graph paper. Each mean value was multiplied by 100 to give values on a 0 to 100 scale, and the zeros were placed on a circle some distance from the centre to give better graphical separation of low values. The 41 variables were plotted on 41 equally-spaced radii, and the key to the variables is given in Table II.

The results previously obtained in the 4, 5, 6 and 7 cluster analyses were analysed in this way, but only a few examples are given here.

Table III shows the original 7 cluster analysis, from which it will be seen that Cluster 1 contains most of the marker cases of carcinoma, and a few cases of leukoplakia. As reported previously, about 35% of the leukoplakia cases placed in this cluster later developed carcinoma. Cluster 2 contains a large group of keratosis and leukoplakia cases with little else, and Cluster 3 contains mainly cases diagnosed as lichen planus.

Fig. 3 shows the polar vector diagrams illustrating the relative importance of each variable in defining the characteristic of each of these clusters.

In Cluster 1 (carcinoma and " severe " leukoplakia) the peak at variable 5 represents acanthosis, and the peak for variable 10 represents micro-organisms (including Candida) in the epithelium. The next two peaks, for variables 12 and 13, represent intra-epithelial keratinization and spongiosis. The high values for variables 18–22 represent chronic inflammatory cells in the epithelium. epithelial cell pleomorphism and hyperchromatism, disturbance in polarity of the basal cells, and enlarged nucleoli in the stratum spinosum. The high values at positions 24–26 represent increased mitotic activity in the prickle and basal cell layers, and abnormal mitotic figures in the prickle cell layer. Finally, the high values for variables 29–35 represent various features of the inflammatory cell infiltration in the connective tissue. In a previous paper (Kramer *et al.*, 1970*b*) we drew attention to the apparent importance of Russell bodies (variable 35) and it is of interest that the original paper by Russell (1890) noted an association between these structures and malignant disease.

One further example will suffice to illus-

TABLE III.—*The 248 Biopsies Divided into 7 Clusters by the Computer on the Basis of the Recorded Histological Features. Each Cluster formed by the Computer has been Analysed to Show the Original Histological Diagnoses*

| Original diagnosis | Number of biopsies | Cluster number 1 | 2 | 3 | 4 | 5 | 6 | 7 |
|---|---|---|---|---|---|---|---|---|
| Carcinoma | 13 | 11 | — | — | — | — | — | 2 |
| Leukoplakia | 60 | 8 | 17 | — | 10 | 7 | 8 | 10 |
| Keratosis | 127 | 1 | 84 | 1 | 11 | 20 | 4 | 6 |
| Lichen planus | 48 | — | 1 | 33 | — | 7 | 3 | 4 |

trate this method of analysis. Cluster 3 in Fig. 3 shows the characteristics of a group consisting almost entirely of cases diagnosed as lichen planus.

The peak for variable 1 shows the common involvement of multiple intraoral sites. Peaks for variables 8, 9 and 11 show the importance of separation of the epithelium from the connective tissue, liquefaction degeneration, and the presence of a stratum granulosum. The high value for variable 18 represents the chronic inflammatory cell infiltration in the connective tissue, whilst the peaks for variables 29, 31 and 33 show that this infiltration is mainly in the superficial part of the connective tissue, it is dense, and it consists mainly of lymphocytes.

So far, reference has been made only to the peaks or high values; in other words, to features that characterize the cluster by being present in almost all cases. However, low values also characterize a cluster: for example, in lichen planus cases of Cluster 3 in Fig. 3, variables 24, 25, 26 and 27 are all at or near zero, and this shows that, typically, increased mitotic activity and abnormal mitoses are not found.

### DISCUSSION

When presented with a new series of cases that had been diagnosed as lichen planus by conventional methods, the computer used weighting factors calculated in a previous investigation and "diagnosed" lichen planus in 36 out of the 41 cases. Of course, the computer was faced with a task that was grossly simplified in terms of diagnostic histopathology, the task being simply to discriminate between leukoplakia and lichen planus. However, this further analysis lends support to the validity of the weighting factors previously calculated for the histological variables.

The data presented in the form of the polar vector graphs show, in quantitative terms, the histopathological characteristics of groups of cases identified as similar by the computer, and therefore placed together in a cluster. When such a cluster consists almost entirely of cases given a single diagnosis by conventional methods, these cases may be regarded as typical. Therefore, the quantitative expression of the histological characteristics of the cluster provides a "fingerprint" of that diagnosis.

Any experienced pathologist can say that, in a given condition, some tissue changes are common, others are less common, and others are rarely seen. The analyses presented here express "common", "less common" and "rare" in quantitative terms for computer formed clusters of cases. It is suggested that data of this type will be necessary if diagnostic histopathology is to become less subjective.

It is also possible that previously unrecognized variations in the histopathological pattern of a disease, or even previously unrecognized diseases, might be identified by these types of analyses.

We are greatly indebted to Professor R. B. Lucas and to Miss L. Lister, who collaborated in the earlier studies which provided part of the data analysed here, and to Mr Michael Clarke for his help with the analytical methods.

### REFERENCES

KRAMER, I. R. H. (1969) Precancerous Conditions of the Oral Mucosa, a Computer-aided Study. *Ann. R. Coll. Surg.*, **45**, 340.

KRAMER, I. R. H., LUCAS, R. B., EL-LABBAN, N. & LISTER, L. (1969) Computer-aided Cluster and Discriminant Analyses of the Histologic Features of Some Oral Mucosal Lesions. *J. dent. Res.*, **48**, 1096.

KRAMER, I. R. H., LUCAS, R. B., EL-LABBAN, N. & LISTER, L. (1970*a*) A Computer-aided Study on the Tissue Changes in Oral Keratosis and Lichen Planus and an Analysis of Case Groupings by Subjective and Objective Criteria. *Br. J. Cancer*, **24**, 407.

KRAMER, I. R. H., LUCAS, R. B., EL-LABBAN, N. & LISTER, L. (1970*b*) The Use of Discriminant Analysis for Examining the Histological Features of Oral Keratosis and Lichen Planus. *Br. J. Cancer*, **24**, 673.

RUSSELL, W. (1890) The Characteristic Organisms of Cancer. The Acidophil Bodies (Fungus Particles). *Br. med. J.*, ii, 1356.

SMITH, C. & PINDBORG, J. J. (1969) *Histological Grading of Oral Epithelial Atypia by the Use of Photographic Standards.* Copenhagen: Department of Oral Pathology, Royal Dental College.